# Regulatory cytokine function in the respiratory tract

**DOI:** 10.1038/s41385-019-0158-0

**Published:** 2019-03-15

**Authors:** William J. Branchett, Clare M. Lloyd

**Affiliations:** 1 National Heart and Lung Institute, Imperial College London, London, United Kingdom; 2 Asthma UK Centre in Allergic Mechanisms of Asthma, Imperial College London, London, United Kingdom

## Abstract

The respiratory tract is an important site of immune regulation; required to allow protective immunity against pathogens, while minimizing tissue damage and avoiding aberrant inflammatory responses to inhaled allergens. Several cell types work in concert to control pulmonary immune responses and maintain tolerance in the respiratory tract, including regulatory and effector T cells, airway and interstitial macrophages, dendritic cells and the airway epithelium. The cytokines transforming growth factor β, interleukin (IL-) 10, IL-27, and IL-35 are key coordinators of immune regulation in tissues such as the lung. Here, we discuss the role of these cytokines during respiratory infection and allergic airway disease, highlighting the critical importance of cellular source and immunological context for the effects of these cytokines in vivo.

## Introduction

The airways face constant assault from the external environment, encountering a mixture of harmless inhaled particles, pathogens, allergens, and pollutants. As immune-mediated tissue damage can compromise gas exchange, pulmonary immunity must be tightly regulated, enabling pathogen clearance and rapid restoration of homeostasis, while avoiding unnecessary, overexuberant, or chronic responses. Aberrant pulmonary immunity is a central feature of asthma,^1^ a chronic inflammatory disease of the airways. Asthma is a complex syndrome that presents as several distinct clinical phenotypes. Many patients display allergic asthma, characterized by a type 2 immune response and sensitization to one or more aeroallergens.^2^ However, much of the global health burden of asthma is due to patients with severe forms of the disease, who respond poorly to conventional therapies (inhaled corticosteroids and bronchodilators) and in whom disease mechanisms are less well understood and can feature non-type 2 immunity.^2^

Excessive pulmonary immune responses can also enhance morbidity and mortality of respiratory infection. Although immune-mediated clearance of pathogens is beneficial to the host, this can occur at the cost of severe immunopathology, such that so-called “tolerance” of the infection, through a more measured immune response, can be preferable.^3,4^ Indeed, retrospective analysis of the highly deadly 1918 pandemic influenza virus, using animal models, strongly suggested that the severity of this viral strain was dependent in part upon on its elicitation of a rapid and excessive host immune response.^5,6^ The balance between activation and regulation of pulmonary immunity is therefore critical for the pathogenesis of both asthma and respiratory infection.

Several cell types are implicated in regulation of immune responses in the lung, including FoxP3^+^ and FoxP3^−^ regulatory T-cell (Treg) subsets,^7^ resident airway macrophages (AMs),^8^ interstitial macrophages (IMs),^9^ dendritic cells (DCs)^10^, and the conducting airway and alveolar epithelia,^11^ highlighting the importance of cell–cell communication in controlling pulmonary immunity. Such cellular interactions in the immune system depend upon signaling mediated by cytokines. In this review, we summarize current knowledge of the most extensively studied immune regulatory cytokines: transforming growth factor β (TGF-β) and interleukins (IL-) 10, 27, and 35, focusing on their involvement in control of immune responses during respiratory infection and allergic airway disease (AAD).

## TGF-β signaling

TGF-β1 is the prototypic cytokine of the TGF-β family (consisting of isoforms 1, 2, and 3; generically referred to here as TGF-β) and is the isoform most widely implicated in immune regulation.^12–15^ Extracellular TGF-β binds to TGF-β receptor type 2 (TGF-βR2), a constitutively active receptor serine/threonine kinase, which recruits and phosphorylates a second serine/threonine kinase, TGF-βR1.^16^ Phosphorylated TGF-βR1 binds and phosphorylates receptor Smad transcription factors Smad2 and/or Smad3, which control gene expression as hetero-oligomers, in partnership with the co-factor Smad4.^17,18^ The TGF-βR complex is widely expressed on both stromal and immune cells and its activation drives diverse gene expression changes that differ substantially depending on the receiving cell type and cross-talk with other contextual signals.^19^ Smad signaling is negatively regulated by the TGF-β-inducible inhibitory Smad, Smad7, which bridges interactions between TGF-βR and the E3 ubiquitin ligase Smurf2 to target the receptor complex for proteasomal degradation,^20^ and protein phosphatase 1, which dephosphorylates TGF-βR1.^21^ TGF-β signaling through non-Smad pathways is also possible.^22^

## TGF-β latency and activation

TGF-β is unusual among cytokines in that its bioactivity is tightly regulated following secretion from the cell. TGF-β isoforms are secreted in complex with a latency-associated peptide (LAP), which prevents interactions with TGF-βR2^23^ and anchors the cytokine to extracellular matrix (ECM) by covalent association with latent TGF-β-binding proteins (LTBPs).^24^ Latent TGF-β can be activated by proteases, heat and acidic pH in vitro.^25,26^ The relevance of these activation mechanisms in vivo are unclear, but it has been suggested that thrombospondin released by activated AMs can position latent TGF-β for activation by the protease plasmin in the airways.^27^ Better understood is the activation of TGF-β1 and TGF-β3 by integrins, which bind an arginine–glycine–aspartate sequence in the LAP to allow release of the active cytokine,^28^ in a manner thought to depend upon mechanical force against the ECM via LTBPs and the cytoskeleton of the integrin-bearing cell.^29,30^ In particular, integrins αvβ6 on epithelial cells^30^ and αvβ8 expressed by leukocytes such as monocytes, macrophages,^31^ DCs^32^, and Tregs^33^ are known to activate TGF-β in vivo. Consequently, although TGF-β is produced by many stromal cell and leukocyte subsets, including effector and regulatory T cells, its bioactivity is precisely regulated to guide its function in vivo.^34^

## TGF-β function in immunity and tissue repair

TGF-β1-null mice die in the first weeks of life from multi-organ immunopathology,^14,15^ underscoring the importance of this cytokine in immune regulation. TGF-β1 has potent and diverse effects on immune responses, which are reviewed in depth elsewhere.^34,35^ In brief, TGF-β1 can suppress Th1 and Th2 cell fates in CD4^+^ T cells, but promote Th9, Th17, and FoxP3^+^ inducible Treg differentiation, in a manner dependent on integration with other cytokine signals.^34^ TGF-β1 also has anti-inflammatory effects on innate leukocytes such as macrophages and natural killer (NK) cells,^35^ illustrating its broad immunoregulatory function. TGF-β also functions at multiple stages of tissue repair and is therefore a master regulator of wound healing and fibrosis.^36^ Appropriate magnitude, timing, and location of TGF-β expression and activation is therefore key to regulating immune responses and restoring tissue integrity, while avoiding fibrosis.

## TGF-β expression and activation in the lung

**Fig. 1:**
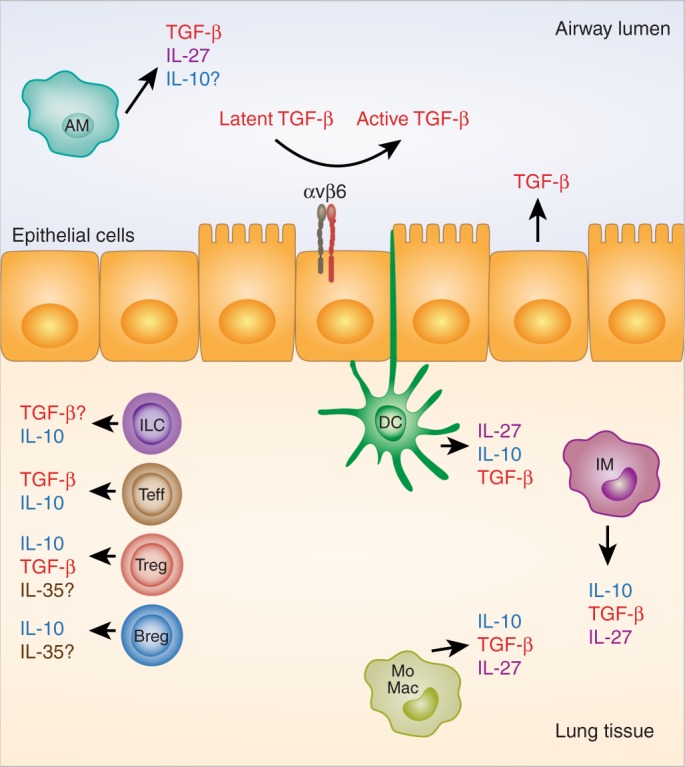
Cellular sources of immune regulatory cytokines in the lung. Transforming growth factor β (TGF-β) is produced by both stromal cells (e.g., bronchial, bronchiolar, and alveolar epithelial cells) and leukocytes in the lung, including resident macrophages, recruited monocyte-derived macrophages (MoMacs), and infiltrating regulatory and effector T cells (Tregs and Teffs). It remains to be seen whether innate lymphoid cells (ILCs) can produce TGF-β in the lung, as they do in the intestine. Integrin αvβ6 on lung epithelial cells is important for activation of latent TGF-β. T cells and interstitial macrophages (IMs) are major IL-10-producing cells in the lung, with expression also reported in dendritic cells (DCs), regulatory B cells (Bregs) and ILCs. Some studies report IL-10 production from airway macrophages (AMs), whereas others suggest that these cells produce little to no IL-10. IL-27 is produced by pulmonary myeloid cells, including AMs, DCs, and likely also resident IMs and recruited MoMacs. IL-35 expression is restricted to Tregs and Bregs and its expression in the lung requires further evaluation

The catastrophic immunopathology that develops in *Tgfb1*^−/−^ mice includes lung inflammation and edema,^14,15^ broadly highlighting the importance of TGF-β1 for regulation of pulmonary immunity. Moreover, αvβ6 integrin, expressed on airway epithelial cells, acts to limit steady state lymphocytic airway inflammation^37^ and emphysema^38^ and promote bleomycin-driven pulmonary fibrosis,^30^ all by local activation of latent TGF-β. TGF-β1 is expressed by diverse cell types in the healthy human and murine lung, including bronchial/bronchiolar and alveolar epithelial cells, endothelial cells, and AMs,^39–41^ with additional TGF-β-producing leukocytes infiltrating the lungs during inflammation or injury^39^ (Fig. 1). The TGF–βR complex is also widely expressed by pulmonary stromal and immune cells,^42,43^ allowing for diverse potential TGF-β signaling events in vivo. Notably, bronchial/bronchiolar epithelial club cells are the major cellular source of bioactive TGF-β1 released into the airway lumen upon inhalation of house dust mite (HDM) allergen or the alarmin IL-33,^39^ or infection with influenza.^44^

## TGF-β in regulation and development of AMs

It is increasingly apparent that TGF-β is crucial for controlling AM phenotype and function. TGF-β1 increases expression of the regulatory receptor CD200R on AMs, ligation of which by CD200 on epithelial cells limits their pro-inflammatory response to influenza infection.^45^ Mice lacking α_V_β_6_ integrin on airway epithelial cells display spontaneous AM activation^46^ and develop emphysema due to overproduction of the AM-derived matrix metalloproteinase (MMP) 12,^38^ suggesting that local TGF-β activity maintains their homeostatic phenotype. Similarly, deletion of TGF-βR2 on myeloid cells led to more severe emphysema during experimental hookworm infection, accompanied by increased MMP activity, a phenotype proposed to depend on dysregulation of lung macrophages in the absence of TGF-β signaling.^47^ AMs are also a major TGF-β1 source in the lung and this TGF-β is required for AMs to induce FoxP3 expression in naive CD4^+^ T cells in vitro.^48^ More recently, TGF-β signaling was shown to be critical for AM development from embryonic precursors during early life, with autocrine TGF-β1 required later in post-natal AM maturation.^41^ Further studies are required to determine whether TGF-β1 derived from AMs regulates immune responses in vivo.

## Protective and pathogenic effects of TGF-β in respiratory infection

**Fig. 2:**
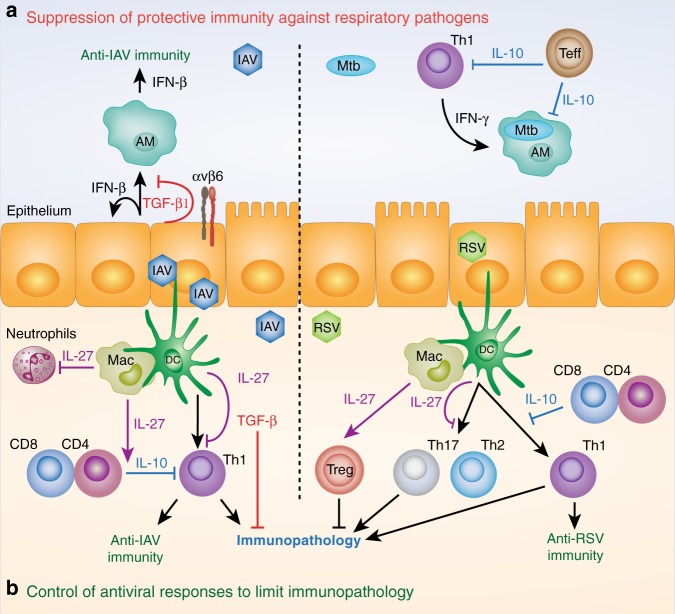
Regulatory cytokine function during respiratory infection. **a** Regulatory cytokine activity can hamper clearance of respiratory pathogens. Influenza A virus (IAV) increases expression of transforming growth factor β (TGF-β) 1 by airway epithelial cells, which is activated by viral neuraminidase and integrin αvβ6. Bioactive TGF-β suppresses interferon (IFN)-β production by epithelial cells and AMs thus, facilitating IAV infection and dissemination. Control of intracellular Mycobacterium tuberculosis (*Mtb*) infection in pulmonary macrophages (including, but not restricted to, AMs) is dependent on activation by IFN-γ and is suppressed by T-cell-derived IL-10. **b** Selected mechanisms of restriction of immunopathology during respiratory viral infection are shown. During IAV infection, T-cell-derived IL-10 limits Th1-dependent immunopathology and its expression is enhanced by myeloid-derived IL-27. IL-27 can also suppress the Th1 response to IAV independently of IL-10 and limit infiltration of innate cells such as neutrophils. TGF-β is required to limit T-cell-dependent immunopathology late in IAV infection. During respiratory syncytial virus (RSV) infection, IL-27 favors protective Th1 immunity over pathogenic Th2 and Th17 cell responses and T-cell-derived IL-10 controls Th1 responses to limit immunopathology. IL-27 can also limit RSV immunopathology by augmenting the suppressive capacity of regulatory T cells (Tregs)

TGF-β is a key regulator of immune responses to influenza infection. Influenza A virus (IAV) drives enzymatic and integrin-mediated release of bioactive TGF-β1 into the airway lumen,^44,46,49–51^ including cleavage of latent TGF-β1 by the IAV neuraminidase,^51^ as well as de novo synthesis from club cells.^44^ Antibody blockade of all TGF-β isoforms during primary influenza infection enhances morbidity and mortality from 6 days post inoculation, suggesting that TGF-β is required to control the cell-mediated adaptive immune response, which peaks around this timepoint^49^ (Fig. 2b). Conversely, TGF-β can also aid generation of protective adaptive immunity to influenza. Lack of TGF-β signaling to CD4^+^ T cells impairs their differentiation into T follicular helper cells during influenza infection, likely owing to a requirement for TGF-β to limit IL-2 signaling to the differentiating T cells and suppress Th1 fate.^52^ In this context, TGF-β is required to mount optimal influenza-specific mucosal IgG and IgA responses.^52^

In contrast to global blockade, targeting of specific sources of TGF-β activity using either club cell-specific TGF-β1 knockout^44^ or αvβ6 integrin knockout mice^46^ does not enhance immunopathology. Instead, these temporally- and spatially-specific TGF-β sources were shown to suppress the antiviral interferon (IFN)-β response to influenza in airway epithelial cells and macrophages^44,46^ (Fig. 2a). In both studies, diminished TGF-β bioactivity resulted in reduced viral burden or dissemination, a dampened downstream immune response and less severe immunopathology and morbidity.^44,46^ Dampening of IFN-β production by TGF-β has also been demonstrated in vitro in rhinovirus infected human bronchial epithelial cells (specifically TGF-β2)^53^ and in macrophages infected with RSV,^54^ suggesting that this may be a common feature of respiratory viral infections capable of driving release of bioactive TGF-β.

In addition to its effects on immunity, TGF-β activation during influenza infection promotes epithelial cell apoptosis and collagen deposition, dependent on Smad3 and αvβ6 integrin, suggesting that TGF-β could contribute to the potential pro-fibrotic sequelae of influenza infection.^50^ TGF-β from diverse cellular sources therefore acts via context-specific mechanisms to govern the outcomes of influenza infection.

The function of TGF-β during other respiratory infections has been less extensively studied. In respiratory syncytial virus (RSV) infection, where Th1 responses are generally protective and favor viral clearance, while Th2 and Th17 responses are implicated in immunopathology, particularly in infants,^55^ TGF-β has been proposed to impair the neonatal adaptive immune response. Cord blood-derived DCs produced more TGF-β than their adult counterparts and drive less protective IFN-γ production and more pathogenic IL-4 and IL-17A from autologous T cells in co-culture.^56^ TGF-β may also favor RSV infection of airway epithelial cells, since TGF-β increased RSV replication in human bronchial epithelial cells and the alveolar epithelial A549 cell line.^57^ Enhanced viral replication in these cells was accompanied by cell cycle arrest, which alone was sufficient to increase viral proliferation, offering a plausible mechanism of action for TGF-β.^58^ Unlike influenza, RSV infection does not drive club cell-dependent TGF-β1 release into the airways and club cell-restricted TGF-β1 knockout did not impact the RSV-driven IFN-β response or immunopathology in vivo.^44^ However, αvβ6 integrin knockout mice were protected from lethal infection with Sendai virus or *Streptococcus pneumoniae*, as well as influenza, suggesting that integrin-mediated TGF-β activation may limit protective immunity against diverse respiratory pathogens.^46^

## Protective and pathogenic effects of TGF-β in allergic airway disease

**Fig. 3:**
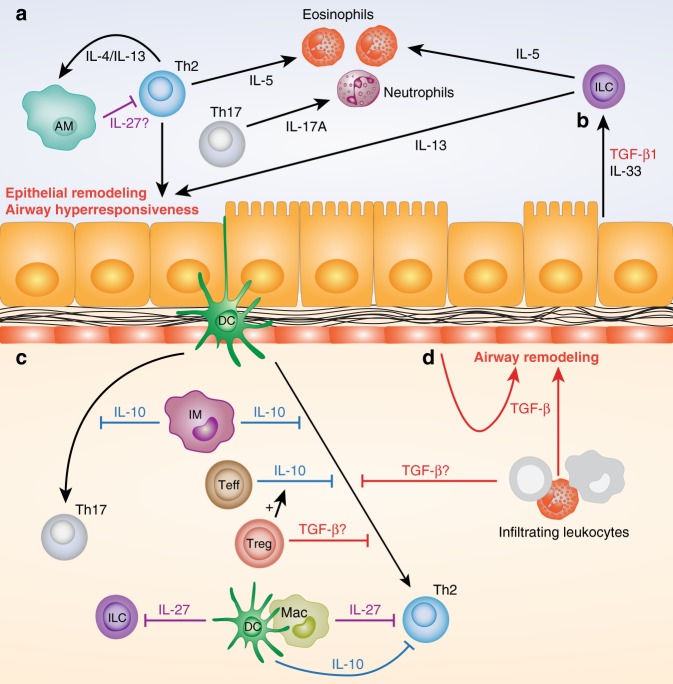
Cytokine regulation of allergic lung inflammation. **a** Type 2 cytokines (IL-4, IL-5, and IL-13) drive multiple pathogenic features of allergic airway disease (AAD), including eosinophilic inflammation, structural changes to epithelium, and activation of macrophages. Airway macrophages (AMs) are required to limit the pathogenic Th2 response to allergen, possibly via IL-27. **b** Epithelial club cells release transforming growth factor β (TGF-β) 1 following allergen or IL-33 exposure, which acts in concert with IL-33 to drive type 2 innate lymphoid cell (ILC2) recruitment and activation. **c** IL-10 from interstitial macrophages (IMs) can limit both Th2 and Th17 responses to allergen, depending on the experimental model. Effector T-cell (Teff) -derived IL-10 limits Th2-cell responses to allergen, and this IL-10 expression can be enhanced by regulatory T cells (Tregs). TGF-β also limits Th2-cell responses to allergen, although the functionally dominant source (Tregs, infiltrating leukocytes, or other cell types) is unclear. Myeloid-derived IL-27 can limit both ILC2 and Th2-cell responses to allergen and dendritic cell (DC)-derived IL-10 has been suggested to promote tolerance and suppress Th2 responses. **d** TGF-β, potentially derived from leukocytes and stromal cells, may also promote remodeling of the epithelium, mesenchymal cells, and extracellular matrix of the airway wall

Allergic asthma and murine models of AAD feature chronic inflammation, airway hyperresponsiveness (AHR) and progressive structural changes to the airway wall, termed remodeling.^1^ As TGF-β signaling is activated following airway allergen challenge in both mice and humans^39,43^ and is widely implicated in both immune regulation^35^ and tissue repair,^59^ it is a cytokine of great interest for studies of the etiology of AAD. Several studies support a broad requirement for TGF-β to regulate immunity to inhaled allergens. Heterozygous *Tgfb1*^−/+^ mice with reduced systemic TGF-β1 expression display enhanced type 2 lung inflammation in a model of peripheral sensitization and airway challenge with the model allergen ovalbumin,^60^ consistent with the known ability of TGF-β to limit Th2 cell generation^61^ (Fig. 3c). Conversely, adoptive transfer of ovalbumin-specific CD4^+^ T cells engineered to overexpress TGF-β1 was sufficient to reverse type 2 inflammation and AHR in an antigen-specific manner.^62^

However, studies using integrin knockout mice to prevent activation of specific TGF-β sources have suggested roles for TGF-β in promoting AHR.^63,64^ Epithelial αvβ6 expression was required for TGF-β-dependent modulation of mast cell proteases, which favored airway contractility in mice.^63^ Similarly, activation of TGF-β by αvβ8 integrin on DCs was required for production of IL-17A by CD4^+^ αβ T cells following peripheral allergen sensitization and airway challenge in mice, with IL-17A shown to promote contraction of murine and human airway smooth muscle cells (ASMCs) in vitro.^64^ In addition, overnight TGF-β1 exposure has been shown to increase contractility of human ASMCs and to desensitize these cells to relaxation by β_2_ adrenergic receptor agonists,^65,66^ suggesting that TGF-β itself may directly promote AHR in some contexts. TGF-β also has potential to drive pathogenic airway structural changes, since hyperactivation of TGF-β signaling in the conducting airway epithelium by overexpression of Smad2 enhanced airway remodeling and AHR in mice exposed to inhaled ovalbumin without prior sensitization,^67^ or mice repeatedly administered inhaled HDM.^68^ Notably, these pathogenic effects of Smad2 overexpression occurred without increasing the type 2 immune response to allergen.^68^ Thus, TGF-β can independently influence the inflammatory, airway dysfunction and remodeling components of AAD (Fig. 3).

The balance between anti-inflammatory and pro-remodeling effects of TGF-β in AAD appear to depend upon the route, and therefore cellular mechanisms, of allergic sensitization. Blockade of all TGF-β isoforms after disease onset in a model of peripheral sensitization and airway challenge with ovalbumin reduced airway remodeling, without affecting type 2 immunity.^69^ In contrast, therapeutic administration of the same blocking antibody in a mouse model where AAD is elicited by repeated mucosal exposure to HDM did not reduce airway remodeling, but instead enhanced airway eosinophilia and AHR, indicative of impaired immune regulation.^70^

Conflicting roles of TGF-β between AAD models are likely to reflect differential utilization of distinct cellular sources of TGF-β, depending on the precise nature of the immune response to allergen, which in turn is governed by the type of allergen and route of exposure. Cell type-specific functions of TGF-β1 in AAD are supported by experiments using a club cell-specific TGF-β1 knockout mouse, which revealed a role for bronchiolar epithelial-derived TGF-β1 in promoting type 2 innate lymphoid cell (ILC2) recruitment and activation, in concert with IL-33^39^ (Fig. 3b). Conditional knockout of additional cellular sources of TGF-β will likely reveal more distinct functions of this cytokine in AAD. As TGF-β is expressed by both leukocyte and stromal cell populations in human lung^40^ and appears to be released and activated following allergen challenge of asthmatic subjects,^43,71^ it is likely that diverse, context-specific, TGF-β signaling events are also relevant to pathogenesis of human asthma.

## IL-10 signaling

IL-10 signals via the heterodimeric IL-10 receptor (IL-10R), comprising the ligand-binding subunit IL-10Rα^72^ and its signaling partner IL-10Rβ, which also forms part of the receptors for other cytokines such as IL-22.^73,74^ IL-10R is broadly expressed by hematopoietic cells and IL-10Rα can be upregulated on monocytes, neutrophils, and certain CD4^+^ T cell subsets upon activation to increase IL-10-responsiveness.^75,76^ Together, IL-10Rα and IL-10Rβ trigger transphosphorylation of associated Janus kinases Jak1 and Tyk2, which allow docking and phosphorylation of combinations of the signal transducer and activation of transcription (STAT) transcription factors, STAT1, STAT3, and STAT5, that mediate gene expression changes.^77,78^ These changes include induction of anti-inflammatory suppressor of cytokine signaling 3 and suppression of production of pro-inflammatory mediators such as IL-6 and tumor necrosis factor α.^79,80^

## Cellular sources of IL-10

IL-10 was initially classed as a type 2 cytokine, owing to its production by Th2 cell clones and ability to inhibit IFN-γ production by Th1 cells.^81^ However, it is now known that IL-10 can also be produced by diverse leukocyte populations, including Th1 and Th17 effector CD4^+^ T-cell (Teff) subsets, FoxP3^+^ and FoxP3^−^ Tregs, CD8^+^ T cells, NK cells, monocytes, macrophages, DCs, and a subset of regulatory B cells (Bregs), as reviewed by Saraiva and O’Garra^82^ (Fig. 1). Recently, an IL-10 and TGF-β-producing ILC population was shown to regulate intestinal inflammation.^83^ Similarly, a small population of IL-10^+^ ILCs was detected in nasal tissue of patients with chronic rhinosinusitis with nasal polyps and in lungs of mice following repeated HDM inhalation, and retinoic acid was shown to induce IL-10 production from ILC2s expanded from human blood.^84^ Further studies will be required to evaluate the importance of IL-10^+^ ILCs in respiratory health and disease.

## IL-10 immune regulatory functions

IL-10 is a potent suppressor of antigen-presenting cell (APC) function, limiting both antigen presentation and cytokine production by macrophages and DCs and thereby preventing Th1 cell polarization by these cells in vitro.^85–87^ IL-10 can also directly act on CD4^+^ T cells, as experiments in which IL-10R is deleted or non-functional on CD4^+^ T cells have demonstrated dysregulation of pathogenic Th17^88^ and Th2^89^ cells in the absence of direct IL-10 signaling. The crucial anti-inflammatory function of IL-10 is well illustrated by the early discovery that spontaneous Th1-driven colitis occurs in global IL-10 knockout (*Il10*^−/−^) mice.^90,91^ However, IL-10 can regulate diverse immune responses during infection, autoimmunity, allergy, and cancer, as reviewed elsewhere.^92^ Here, we focus on IL-10 regulation of immunity during infection and allergic inflammation in the lung.

## IL-10 suppression of pathogenic and protective immunity during respiratory infection

Global *Il10*^−/−^ mice and IL-10Rα blocking antibodies have been used to demonstrate the importance of IL-10 for regulation of immune responses in the lung. IL-10Rα blockade in mice increases the severity of respiratory disease in mice infected with influenza or RSV,^93–95^ with similar results obtained in RSV-infected *Il10*^−/−^ mice.^95^ Importantly, with both viruses, increased severity was not accompanied by increased viral burden, but featured a heightened pulmonary Th1 response,^93–95^ indicative of a failure to regulate adaptive immunity and tolerate infection in the absence of IL-10 signaling (Fig. 2b). Conversely, the anti-inflammatory effects of IL-10 can be detrimental to the clearance of pulmonary pathogens, such as the intracellular bacterium *Mycobacterium tuberculosis* (*Mtb*).^96^ Protective immunity to mycobacterial infection is dependent on an effective Th1 response and IFN-γ activation of *Mtb*-infected pulmonary macrophages, which can be impaired by IL-10^85,97,98^ (Fig. 2a). Accordingly, increased levels of IL-10 are present in the airways of patients with tuberculosis^99,100^ and correlate with pulmonary *Mtb* antigen burden,^101^ suggesting that IL-10 induction in the host is a strategy of *Mtb* survival. Indeed, a highly pathogenic *Mtb* strain has been identified with a heightened ability to induce IL-10 in macrophages.^102^ Work in *Mtb* illustrates the importance of measured IL-10 function in the lung, to restrict immunopathology without compromising immunity to pathogens.

Understanding of pulmonary IL-10 function is complicated by the numerous potential cellular sources of this cytokine.^82^ However, T cells are one clearly important source of IL-10 in the lung. In the context of respiratory viral infection, T-cell-restricted deletion of IL-10 recapitulates the effects of global IL-10 blockade by augmenting Th1-dependent immunopathology during influenza and RSV infections in mice,^93,94^ with both CD4^+^ and CD8^+^ T cells producing IL-10 in these models (Fig. 2b). In addition, CD4^+^ Teffs were shown to be the functionally dominant source of IL-10 in restricting host immunity to *Mtb* infection in mice^103^ (Fig. 2a). B cells are less well characterized than T cells as an IL-10 source during respiratory infection, but IL-10^+^ Bregs have been shown to limit pathogenic pulmonary Th1 and Th17 responses in a murine model of *Pneumocystis* infection.^104^

## IL-10 regulation of allergic airway disease

Despite its well-established role in suppression of Th1 immunity, IL-10 can regulate pulmonary Th2 responses to allergens in certain contexts, as demonstrated by the increased type 2 cytokine production and airway eosinophilia in *Il10*^−/−^ mice subjected to systemic sensitization and airway allergen challenge protocols.^105,106^ Indeed, following systemic sensitization and airway challenge with HDM, direct signaling of IL-10 to Th2 cells is required to limit their survival and dampen allergic airway inflammation.^89^ However, IL-10 does not exclusively suppress type 2 responses to allergen in the lungs. In a model of AAD driven by repeated HDM inhalation, global IL-10 knockout resulted in heightened pulmonary IL-17A expression and neutrophilia,^107^ suggesting that the role of IL-10 in experimental AAD models differs depending on the route of allergen exposure and underlying disease mechanisms.

As in respiratory infection, CD4^+^ T cells are a critical source of IL-10 during AAD. In a model of resolution of allergic inflammation by transfer of CD25^hi^ Tregs to sensitized mice, dampening of the type 2 inflammatory response to inhaled ovalbumin was dependent on induction of IL-10 expression in recipient CD4^+^ T cells^108^ (Fig. 3c). Similarly, transfer of CD4^+^ T cells overexpressing IL-10 to the lungs was sufficient to reverse airway inflammation and hyperresponsiveness in a similar model.^109^ Induction of IL-10 production by CD4^+^ T cells was also essential for the efficacy of peptide immunotherapy in a murine model of cat allergen-driven airway inflammation^110^ and is associated with effective immunotherapy with grass pollen allergens in patients with seasonal allergic rhinitis (SAR),^111,112^ highlighting the potential for T helper cell-derived IL-10 to limit pathogenic immune responses to aeroallergens. Although IL-10^+^ Bregs have been observed in humans during allergen exposure-driven tolerance to bee venom and suppress antigen-specific proliferation of T cells from these subjects,^113^ it currently remains unclear whether IL-10^+^ Bregs are associated with tolerance to aeroallergens. However, passive transfer of splenic IL-10^+^ Bregs to ovalbumin-sensitized mice was sufficient to suppress allergic airway inflammation,^114^ indicating that these cells can regulate allergic inflammation in vivo if present in sufficient numbers.

Non-lymphoid sources of IL-10 have also been suggested to regulate the airway response to allergens. IMs produce IL-10 at steady state and in response to pathogen-associated molecular patterns^9,115^ (Fig. 1) and can regulate immune responses in several models of AAD when transferred to the lungs or expanded with the TLR9 agonist CPG^9,107,115^ (Fig. 3c). However, the importance of endogenous IMs as an IL-10 source in limiting allergic sensitization and inflammation is unclear from these studies. DC production of IL-10 upon exposure to harmless airborne antigens has also been suggested as a mechanism of inhalation tolerance^116^ (Figs. 1 and 3c). IL-10 expression has been demonstrated in isolated AMs from asthmatic patients and was increased following inhaled corticosteroid treatment^117,118^ (Fig. 1). However, IL-10 production by AMs was substantially lower than peripheral blood monocytes under the same ex vivo stimulation conditions,^118^ raising questions as to the in vivo relevance of AM-derived IL-10. Accordingly, in mice, AMs show minimal steady state IL-10 production capacity when compared with IMs.^115^ Further mechanistic studies will be required to examine specific endogenous cellular sources of IL-10 in different inflammatory contexts.

Data from clinical studies support an association between insufficient IL-10 function and asthmatic inflammation in humans. Reduced airway IL-10 concentrations and IL-10 production by CD4^+^ T cells have been observed in pediatric asthmatics with severe therapy-resistant disease^119^ and CD4^+^ T cells from steroid-resistant adult asthmatics show diminished IL-10 production in response to in vitro corticosteroid treatment.^120^ Associations between polymorphisms in the *IL10* gene and asthma incidence and severity have also been reported,^121^ although these associations appear to depend on the age and ethnicity of asthmatics.^122^ IL-10 production, particularly from CD4^+^ T cells, is therefore likely to be a key immune regulator in allergic disorders of the human respiratory tract.

## IL-27 signaling

IL-27 is active as a heterodimer of the IL-12-family p28 α chain subunit and the soluble binding receptor Epstein–Barr virus-induced gene 3 (EBI3).^123^ IL-27 signals canonically via a receptor complex of IL-27Rα (also known as WSX-1) and the common co-receptor glycoprotein 130 (gp130),^124^ principally by driving phosphorylation of STATs 1 and 3.^124,125^ IL-27 is expressed mainly by myeloid cells, such as macrophages, monocytes and DCs^123,126^ (Fig. 1). The IL-27 receptor complex is widely expressed on leukocytes,^124^ particularly NK cells and T cells, and its expression can vary with activation state.^126,127^

## IL-27 function in immunity

IL-27 was originally described as a pro-inflammatory cytokine that contributes to type 1 immune responses, because of its ability to induce proliferation and IFN-γ production from naive CD4^+^ T cells in synergy with IL-12 or CD28 co-stimulation,^123^ by increasing expression of both Tbet and the β2 subunit of the IL-12 receptor.^128^ IL-27 can also promote Tbet expression and cytotoxic activity in NK cells^129^ and drive expression of the type 1-associated cytokines IL-12 and IL-18 in human monocytes.^124^ Conversely, IL-27 does not enhance IL-13 production from naive CD4^+^ T cells cultured under Th2-polarizing conditions,^123^ underscoring its potential as a pro-Th1 cytokine.

Despite its clear ability to promote type 1 immunity, in vivo models in which IL-27 signaling is ablated have demonstrated the capacity of IL-27 to restrain potentially pathogenic immune responses. During infection with *Toxoplasma* (*T*.) *gondii*, an intracellular parasite that is cleared by an IFN-γ-dependent type 1 immune response, global or CD4^+^ T cell-restricted absence of IL-27Rα resulted in dysregulated IFN-γ production by T cells.^130^ Regulation of the Th1 response to *T*. *gondii* by IL-27 is thought to occur at least in part due to its suppression of IL-2 production from CD4^+^ T cells.^130,131^ *Il27ra*^−/−^ mice failed to survive *T. gondii* infection despite effectively clearing the parasite,^130^ positioning IL-27 at the center of the balance between pathogen removal and immunopathology in this type 1 setting. IL-27Rα deficiency had similar effects during the type 2 immune response to infection with the helminth *Trichuris* (*T*.) *muris*, resulting in accelerated worm expulsion, but enhanced type 2 cytokine production and intestinal goblet cell immunopathology.^132^ IL-27 can also prevent generation of the Th17 lineage from naive CD4^+^ T cells in vitro^133,134^ and, at least in human samples, partially reduces IL-17A expression from memory CD4^+^ T cells.^133^ IL-27Rα knockout increases the severity of *Leishmania* (*L*.) *major* infection, accompanied by enhanced generation of IL-17A^+^ CD4^+^ T cells and a diminished type 1 immune response,^135^ and dampens the pathogenic Th17 cell response during experimental autoimmune encephalomyelitis (EAE)^136^ and *T. gondii* infection.^137^ Thus, IL-27 can limit type 1, 2 and 3 immune responses in a context-specific manner.

Anti-inflammatory functions of IL-27 can be partially mediated through induction of IL-10. IL-27 induces IL-10 production from naive CD4^+^ T cells, in particular driving generation of a population of IFN-γ^+^ IL-10^+^ ‘Tr1’ regulatory T cells.^138^ IL-27Rα expression on T cells is required for generation of protective IFN-γ^+^ IL-10^+^ CD4^+^ T cells during EAE^139^ and IL-27 can augment IL-10 production from CD4^+^ T cells under Th1- and Th2-, but not Th17-, polarizing conditions,^125^ demonstrating potential for regulation of diverse T helper cell subsets. However, not all anti-inflammatory effects of IL-27 are IL-10-dependent, as exemplified by the unimpaired IL-10 production from T cells of *T. gondii*-infected *Il27ra*^−/−^ mice.^130^ Care must therefore be taken when interpreting results of manipulation of IL-27 signaling on immune regulation to distinguish direct and IL-10-dependent effects.

## IL-27 regulation of immunity during respiratory infection

Several insights into IL-27 function in the lung have come from studies of respiratory infection. IL-27Rα knockout enhanced immunopathology and mortality in influenza-infected mice, accompanied by increased production of IFN-γ and IL-17A by CD4^+^ T cells.^140^ Notably, suppression of IL-17A, but not IFN-γ, production by IL-27 was IL-10-dependent^140^ (Fig. 2b). Pulmonary IL-27 levels peaked late in influenza infection, and delivery of recombinant IL-27 at this stage of infection limited recruitment of inflammatory monocytes, neutrophils, and NK cells into the lung, without affecting the T cell response, suggesting that IL-27 can operate at multiple levels to control the immune response to influenza.^140^ Others have reported the role of IL-27, produced by lung APCs in a type 1 IFN-dependent manner,^141^ in generation of IL-10^+^ CD8^+^ T cells,^142^ a key regulatory population during influenza infection^94^ (see above).

During RSV infection, absence of IL-27Rα signaling increased Th2 and Th17 responses, at the expense of a protective Th1 response (Fig. 2b), resulting in impaired viral clearance and mucus-dominated pathology that could be ameliorated by blockade of IL-17A.^143^ Similarly, in a model of vaccine-exacerbated RSV infection, where prior vaccination results in Th2 and Th17-dominated immunopathology upon subsequent natural infection, co-administration of plasmids driving IL-27 overexpression dampened the memory Th2 and Th17 responses to RSV, without impairing or enhancing the protective Th1 response, altogether resulting in substantially reduced pathology upon subsequent infection.^144^ These studies suggest that IL-27 can control the balance of protective and pathogenic T helper cell subsets during RSV infection. Using a different approach, Pyle and colleagues have demonstrated a role for IL-27, induced by early IL-6 signaling, in promoting maturation and suppressive capacity of FoxP3^+^ Tregs during primary RSV infection, independently of IL-10^145^ (Fig. 2b).

The immunosuppressive capacity of IL-27 also carries potential to impair pathogen clearance. When administered from the beginning of influenza infection, recombinant IL-27 increased the severity of weight loss and impaired viral clearance, likely by interfering with innate immunity.^140^ Similarly, IL-27 was identified as a factor underlying impaired clearance of respiratory *Pseudomonas aeruginosa* infection in a murine model of sepsis, in part by suppressing the antibacterial activity of AMs.^146^ Timely and measured activity of IL-27 is therefore critical during respiratory infection, to limit immunopathology without compromising pathogen clearance.

## IL-27 regulation of allergic airway disease

In a model of peripheral sensitization and airway challenge with ovalbumin, IL-27Rα deficiency increased pulmonary type 2 cytokine levels, mucus hypersecretion and AHR. Importantly, this enhancement of type 2 immunity was not simply due to lack of cross-inhibition by IL-27-dependent Th1 cells, since the ovalbumin-specific Th1 response was also increased in these mice.^147^ In experiments using EBI3^−/−^ mice, lack of EBI3 was shown to similarly enhance the eosinophilic type 2 immune response to ovalbumin where mice were sensitized via the airways using low dose lipopolysaccharide (LPS), a model which generates a negligible type 1 immune response.^148^ However, EBI3 knockout also enhanced eosinophilia and type 2 cytokine production upon ovalbumin challenge in an otherwise IFN-γ and neutrophil-dominated model, where mice were sensitized using high dose LPS as an adjuvant.^148^ In this context, augmentation of the type 2 immune response was accompanied by decreased IFN-γ levels and neutrophilia,^148^ potentially reflecting loss of Th1–Th2 cross-inhibition. Although conclusions on IL-27 function based on EBI3 knockout must be interpreted with caution, as this subunit also constitutes part of the IL-35 heterodimer (discussed later),^149 ^these data collectively support a role for IL-27 in regulating type 2 immunity during AAD (Fig. 3c). However, differences in phenotype between disease models suggest that the mechanisms of suppression may differ depending on the precise nature of the immune response to allergen, as determined by factors such as route of allergen exposure and the adjuvant effects of allergen components, such as LPS.

The precise mechanisms by which IL-27 regulates immunity during AAD are not fully understood. In vivo AM depletion, which enhances the type 2 immune response to subsequent inhalation of allergen or IL-13, was accompanied by reduced levels of IL-27, but not IL-10 or TGF-β, in lung tissue, suggesting that AMs may be a local source of regulatory IL-27 during AAD^150^ (Fig. 3a). IL-27 regulation of type 2 immunity may in part be mediated by suppressive effects on ILC2s (Fig. 3c). IL-27 is one of several STAT1-activating cytokines shown to suppress ILC2 proliferation and cytokine production and administration of recombinant IL-27 to the airways suppresses ILC2-dependent inflammation induced by inhalation of the fungal allergen *Alternaria alternata*.^151^ IL-27 appears to act directly on ILC2s, as these cells express IL-27Rα^151^ and IL-27Rα-deficient ILC2s outcompeted wild type cells in a mixed bone marrow chimaera system where IL-27 was administered to suppress type 2 inflammation driven by the protease papain.^152^ It is notable that both *Alternaria* and papain are particularly potent inducers of ILC2s in vivo and the contribution of IL-27 effects on ILC2s, CD4^+^ T cells and other cell types in suppression of allergic inflammation is likely to depend upon the relative contributions of these cells to pathogenesis in specific contexts.

## Pathogenic effects of IL-27 on lung function

IL-27 can also be pathogenic in the context of AAD. IL-27 expression is increased in pulmonary macrophages following RSV exacerbation of a mouse model of peripheral sensitization and airway challenge to ovalbumin, accompanied by corticosteroid-resistant lung pathology and IFN-γ-dependent AHR.^153^ In this model, antibody blockade of IL-27 reduced AHR, without influencing pulmonary viral load or IFN-γ levels,^153^ supporting an alternative role for IL-27 in viral exacerbation of AAD, independent of its ability to enhance IFN-γ production by CD4^+^ T cells. A potentially pathogenic role for IL-27 has also been suggested in chronic obstructive pulmonary disorder patients, in whom sputum IL-27 levels are elevated compared with smokers and healthy controls and inversely correlate with lung function.^154^ Moreover, IL-27 promotes expression of the pro-inflammatory chemokine CXCL10 from human bronchial epithelial cells.^154^ Identification of the contextual factors favoring regulatory and pro-inflammatory functions of IL-27 will help understand its role in inflammatory lung disease.

## IL-35 signaling

IL-35 was initially discovered in FoxP3^+^ Tregs, on the basis of their high expression of EBI3, but not IL-27p28, indicative of an alternative binding partner for EBI3 in these cells. Instead, EBI3 was shown to pair with the IL-12α subunit to form IL-35.^149^ IL-35 is preferentially expressed by FoxP3^+^ Tregs and not naive or effector CD4^+^ T cells (Fig. 1) and its expression is required for optimal suppressive activity of Tregs in vitro and in vivo.^149^ IL-35 was initially demonstrated to signal to CD4^+^ T cells, not only via a unique heterodimer of the IL-12R β2 chain and gp130, activating STAT1 and STAT4, but also via homodimers of each component, with distinct signaling outcomes.^155^ IL-35 can also signal to B cells through yet another distinct receptor complex, a heterodimer of IL-12Rβ2 and IL-27Rα, which signals via STAT1 and STAT3, independently of gp130.^156^ This non-classical and variable receptor chain usage is thought to increase the versatility of IL-35 function.^155^

## Il-35 regulation of immunity

IL-35, but not IL-12α or EBI3 alone, suppresses Teff proliferation in vitro^149^ and suppresses Th17 cell generation in vitro and during experimental arthritis in vivo.^157^ IL-35 can also drive differentiation of an IL-35^+^ CD4^+^ Treg population, termed iTr35 cells,^158,159^ when signaling via the IL-12Rβ2-gp130 heterodimer.^155^ A population of FoxP3^+^ IL-35^+^ Tregs exists in secondary lymphoid organs at steady state^160^ and can expand in the gut during helminth infection.^158^ These IL-35^+^ Tregs are distinct from IL-10^+^ FoxP3^+^ Tregs and the two subsets have additive suppressive effects on intestinal inflammation in vivo.^160^ In addition to Tregs, Breg populations have been identified as an IL-35 source (Fig. 1), particularly CD138^+^ plasma cells.^161^ IL-35 expression from B cells limits the pathogenic T-cell response during EAE and B-cell-restricted IL-35 knockout enhanced immunity to *Salmonella* infection.^161^ As with CD4^+^ T cells, IL-35 signaling to B cells suppresses their proliferation and induces expression of both IL-35 and IL-10, generating Bregs that suppress autoimmunity in vivo.^156^

## IL-35 expression in respiratory infection

Relatively little is known about IL-35 function in respiratory infection compared with the other cytokines covered in this review. IL-35 levels are increased in both lungs of mice and serum of humans following influenza infection and its expression is further enhanced in influenza-infected mice following secondary pneumococcal infection.^162^ However, the functional relevance of this IL-35 to disease severity has not been determined, necessitating further mechanistic studies in this context.

## IL-35 suppression of allergic airway disease

In a model of AAD in which a memory Th2 cell line specific for a HDM antigen was transferred into mice prior to airway antigen challenge, pulmonary overexpression of IL-35 was sufficient to suppress type 2 cytokine production and eosinophilia.^163^ Similar results were obtained where IL-35 was expressed in the airways using an adenoviral vector, throughout a model of peripheral sensitization and airway challenge with ovalbumin, concomitant with suppression of IL-17A production and increased numbers of pulmonary FoxP3^+^ Tregs.^164^ Although these studies demonstrate the capacity for local IL-35 to dampen allergic inflammation in the lung, further work will be required to determine the importance of endogenous IL-35 in this context. In a model of AAD elicited by airway sensitization to ovalbumin using LPS as an adjuvant, which drives a Th17-dominated disease to which mice become tolerant following repeated airway ovalbumin exposures, tolerance was dependent upon expansion of a population of ICOS^+^ FoxP3^+^ Tregs. These Tregs suppressed IL-17A production from Teffs in vitro and expressed high levels of both IL-35 subunits, deletion of either of which was sufficient to break tolerance following repeated ovalbumin inhalation.^165^ Thus, IL-35 may suppress pulmonary Th17 responses to allergen.

Despite the limited study of IL-35 function in AAD, expression of both *EBI3* and *IL12A* genes have been reported to be reduced in peripheral blood mononuclear cells from pediatric and adult allergic asthma patients compared with controls, accompanied by decreased plasma IL-35 levels, which inversely correlated with plasma concentrations of the type 2 cytokine IL-4.^166,167^ These findings suggest that insufficient IL-35 production may be a feature of aberrant type 2 immunity in allergic asthma. Accordingly, IL-35 has recently been shown to suppress grass pollen-specific Th2 cell responses in cells from SAR patients and to suppress production of IL-5 and IL-13 by ILC2s from these individuals.^159^ Moreover, IL-35^+^ Tregs were less frequent in peripheral blood of SAR patients, but were elevated with sublingual allergen immunotherapy, inversely correlating with symptom scores.^159^ Although these studies used peripheral blood cells as a surrogate for those from tissue, they suggest a role for IL-35 in regulating the type 2 immune response to grass pollen in the upper airways.

## Conclusions and perspectives

The respiratory tract represents a unique interface with the external environment, at which effective but tightly regulated immune responses are critical to protect against infection while minimizing disruption of the intricate pulmonary architecture. Regulatory cytokines, produced by distinct regulatory B/T-cell populations (IL-35), myeloid cells (IL-27), or by diverse cellular sources (TGF-β and IL-10), are an effective means of fine-tuning immune responses. This “division of labor” across several cytokines and cell types allows regulation of immune response at multiple levels in tissues such as the lung.

Regulatory cytokines present a double-edged sword in respiratory infection, limiting immunopathology, while potentially compromising pathogen clearance (Fig. 2). Characterization of mechanisms underlying the balance between regulatory and pro-inflammatory cytokines will be important in understanding the role of cytokine regulation in tolerance and clearance of respiratory infection. Such mechanisms are well exemplified by the potentially pathogenic role of type 1 IFNs in *Mtb* infection, via induction of IL-10 and dampening of type 1 immunity.^96^ In particular, it will be important to understand mechanisms by which pathogens subvert host regulatory cytokines to favor their own survival, as described for IL-10 in *Mtb*^102^ and TGF-β in influenza^44,46^ (Fig. 2a).

Regulatory cytokines are of great therapeutic interest in allergic disorders of the respiratory tract, such as SAR and asthma, as their induction, particularly in CD4^+^ T cells, is associated with successful immunotherapy in the clinic and in mouse models of tolerance induction to aeroallergens.^110,168^ However, asthma is a highly heterogeneous syndrome, with many clinically and etiologically distinct forms that are currently less effectively treated than “traditional” type 2-dominated allergic asthma.^2^ Much of the understanding of immunological mechanisms of asthma has derived from animal models of AAD, utilizing either peripheral sensitization followed by airway challenge, often with the inert protein ovalbumin, or repeated inhalation of “true” allergens such as HDM or *A. alternata*.^169,170^ However, the contrasting effects of IL-10 deficiency,^105–107^ EBI3 knockout^148^, and TGF-β blockade^69,70^ in different mouse AAD models point to potential for distinct utilization of regulatory cytokines depending on the nature of allergen, the route of exposure and, consequently, the different cellular mechanisms involved in the response.

Such context-specific cytokine function in AAD models underscores perhaps the most important message from this review; that the function of regulatory cytokines varies depending on their cellular source, the nature of the ongoing immune response and cross-talk with other cytokine signals. It is also notable that, although we have discussed four cytokines individually in this review, these cytokines will of course be simultaneously present in certain contexts and are likely to influence the expression and functionality of each other. Studies dissecting roles of different cellular cytokine sources in experimental murine models are likely to be of utmost importance in guiding rational design of therapies based on immune regulation and/or tolerance. Combining such studies with analysis of specific cell populations in human clinical samples will greatly aid understanding of the crucial balance between regulatory and pro-inflammatory cytokine function in the lung.
